# *Lactobacillus johnsonii* N5 Supernatant Protects RPMI-2650 Cells from HDM-Induced Injury in Association with Suppression of the NR4A1–IRE1–XBP1-Mediated Unfolded Protein Response

**DOI:** 10.3390/metabo16090634

**Published:** 2026-08-31

**Authors:** Wei Ma, Yufei Chen, Yaxin Feng, Long Yuan, Haoyu Liu, Yangzi Sun, Zixiang Xu, Demin Cai, Haiyong Sun

**Affiliations:** 1 Department of Otolaryngology, Head and Neck Surgery, Northern Jiangsu People’s Hospital, Yangzhou 225001, China; ybdd1980@163.com; 2 The Yangzhou Clinical Medical College, Xuzhou Medical University, Yangzhou 225001, China; fyx18346642866@163.com (Y.F.); 18952171342@163.com (Y.S.); 3 Northern Jiangsu People’s Hospital Affiliated to Yangzhou University, Yangzhou 225001, China; cyf202137@163.com (Y.C.); a1079904149@163.com (Z.X.); 4 Laboratory of Animal Physiology and Molecular Nutrition, Jiangsu Key Laboratory of Animal Genetics, Breeding and Molecular Design, Yangzhou University, Yangzhou 225009, China; yuanlong0715@foxmail.com (L.Y.); haoyu.liu@yzu.edu.cn (H.L.); 5 Jiangsu Interdisciplinary Center for Zoonoses and Biosafety, Yangzhou University, Yangzhou 225009, China; 6 Guangling College, Yangzhou University, Yangzhou 225009, China

**Keywords:** *Lactobacillus johnsonii* N5, HDM, UPR, human nasal epithelial carcinoma cells, allergic rhinitis, probiotic

## Abstract

**Highlights:**

**What are the main findings?**

- *Lactobacillus johnsonii* N5 supernatant markedly suppresses the NR4A1–IRE1–XBP1 signaling axis.
- *Lactobacillus johnsonii* N5 supernatant markedly attenuates HDM-induced unfolded protein response.

**What are the implications of the main findings?**

- The NR4A1–IRE1–XBP1 axis is identified as a pivotal mechanism responsible for HDM-induced nasal epithelium damage, presenting a novel molecular target for therapeutic intervention in allergic rhinitis and other allergic airway disorders.
- The supernatant of *Lactobacillus johnsonii* N5 shows potential as a postbiotic approach for addressing HDM-induced allergic inflammation, providing a safer option than live probiotics, especially for immunocompromised individuals, by maintaining immunomodulatory properties without the hazards linked to viable microorganisms.

**Abstract:**

**Background/Objectives**: House dust mites (HDMs) are significant allergens that cause damage to nasal epithelial cells and have a role in the pathophysiology of allergic rhinitis. *Lactobacillus johnsonii* N5 supernatant (N5sup), a probiotic strain, exhibits possible protective properties; nevertheless, the molecular processes involved remain unclear. **Methods**: RPMI-2650 human nasal epithelial carcinoma cells were subjected to treatment with HDM, N5sup, or a combination of both. **Results**: The HDM stimulation induced a certain degree of transcriptional alterations. Compared with the control group, a total of 221 differentially expressed genes were identified in HDM-treated cells, which were mainly enriched in the IRE1–XBP1 branch of the unfolded protein response (UPR) pathway. Comprehensive investigation demonstrated that HDM increased the NR4A1–IRE1–XBP1 signaling pathway, characterized by elevated expression levels of *NR4A1*, *ERN1* (IRE1), and *XBP1*. Both qRT-PCR and Western blotting verified that N5sup strongly inhibited the HDM-induced increase in NR4A1, IRE1, and XBP1. Analysis of the protein–protein interaction network further confirmed a central hub comprising NR4A1, IRE1, and XBP1. **Conclusions**: N5sup supernatant protects RPMI-2650 cells from HDM-induced injury, and this protective effect is associated with the NR4A1–IRE1–XBP1 pathway. These findings suggest that this signaling axis may serve as a potential therapeutic target for probiotic intervention in allergic airway diseases.

## 1. Introduction

House dust mites (HDMs), especially *Dermatophagoides pteronyssinus*, are prevalent aeroallergens and significant triggers of allergenicity, contributing to atopic sensitization in 50–85% of asthma patients [1]. Recent research indicates that 65 to 130 million individuals worldwide are hypersensitive, exhibiting detectable allergen-specific IgE in their serum against HDM [2], which accounts for 15–20% of the population in developed nations [3,4]. In Central Europe, 50% of allergy sufferers are susceptible to dust mites [5]. Individuals with an allergy to house dust mites exhibit several clinical manifestations, including allergic rhinoconjunctivitis, atopic dermatitis, and particularly allergic asthma. The latter is characterized by recurrent episodes of dyspnea and wheeze induced by allergen inhalation, stemming from bronchoconstriction and airway inflammation [6,7]. In addition to genetic factors, early childhood exposure to HDM allergens substantially affects the subsequent development of asthma [8]. HDM generates various proteases with proteolytic activity, resulting in the disruption of the epithelial barrier, subepithelial antigen infiltration, dendritic cell activation, and T helper 2 (Th2) cell differentiation, thereby affecting the ensuing allergic inflammatory cascade and categorizing it as Th2 high asthma [1,9,10]. Moreover, HDM can directly stimulate airway epithelium to produce epithelium-derived cytokines, known as alarmins, including thymic stromal lymphopoietin (TSLP), IL-25, and IL-33, which can provoke both innate and adaptive immune responses and enhance Th2 immunity [10,11,12]. HDM aeroallergens exert a considerable impact on asthma, from initial sensitization to advanced disease progression [10]. The airway epithelium operates on analogous principles: when HDM allergens breach the barrier, the ensuing Th2-dominated immune response forms the foundation for allergic rhinitis and allergic asthma.

The endoplasmic reticulum (ER) is the primary location for the folding and processing of secretory and membrane proteins in the cell. When the demand for protein folding exceeds the endoplasmic reticulum’s capacity due to infection, oxidative stress, or inflammatory mediators, misfolded proteins accumulate in the ER lumen, leading to a condition termed ER stress. The cell responds by activating an evolutionarily conserved signaling pathway termed the unfolded protein response (UPR) [13]. The UPR is facilitated by three transmembrane sensors of the endoplasmic reticulum: *IRE1*, *PERK*, and *ATF6*. In homeostatic conditions, the principal chaperone BiP (GRP78) preserves the inactivity of all three sensors by associating with their luminal domains. When misfolded proteins accumulate, BiP is sequestered to promote normal folding, thereby releasing the sensors to oligomerize and activate their respective downstream cascades [14,15]. The UPR employs a tripartite strategy: (1) temporarily suppressing global protein synthesis via PERK to reduce protein influx, (2) enhancing the ER’s folding capacity by upregulating chaperones through IRE1 and ATF6, and (3) promoting the degradation of irreversibly misfolded proteins through ER-associated degradation (ERAD) [16]. In response to ER stress, IRE1α dimerizes and undergoes trans-autophosphorylation, thereby activating its RNase domain. The activated RNase performs an unconventional cytoplasmic splicing event on *XBP1* mRNA: it excises a 26-nucleotide intron from the unspliced *XBP1* mRNA (XBP1u), and the two exon segments are religated by the tRNA ligase RTCB [17]. The splicing process causes a reading-frame shift that transforms the short, inactive *XBP1u* protein into the active transcription factor *XBP1s* (spliced XBP1). *XBP1s* subsequently migrates to the nucleus and modulates the expression of genes related to endoplasmic reticulum expansion, protein folding, ERAD, and lipid synthesis [18]. *NR4A1*, also known as Nur77 or TR3, is an orphan nuclear receptor that operates as a ligand-independent transcription factor, swiftly activated by diverse stimuli, including oxidative stress, cytokines, and metabolic signals. It is extensively expressed in various tissues and regulates cell differentiation, proliferation, survival, and apoptosis [19,20]. NR4A1 expression is altered in inflamed tissues, including those impacted by rheumatoid arthritis, and its modulation can influence the inflammatory trajectory; however, the specific direction (pro- or anti-inflammatory) appears to depend on the tissue and context [21,22]. Although previous studies have provided mechanistic insights into the IRE1–XBP1 pathway and general information on NR4A1, direct investigations of NR4A1’s specific role in airway epithelial ER stress remain limited.

In the last ten years, the therapeutic landscape of gut microbiota-based medicines has undergone a significant conceptual shift. While live probiotics, defined as viable microorganisms that confer health benefits to the host, have historically been the predominant method, escalating safety concerns regarding the use of live microbial cells, particularly in immunocompromised individuals, have stimulated interest in non-viable alternatives [23]. Two alternatives have emerged—paraprobiotics (inactivated microbial cells) and postbiotics (microbial metabolites and by-products)—both of which retain immunomodulatory capabilities without the risks associated with living organisms [24,25]. *Lactobacillus johnsonii* N5 (N5) has received considerable interest among *Lactobacillus* species for its varied immunomodulatory properties in multiple clinical contexts [26,27]. The N5 strain, originally isolated from heat-stress-resistant weaned piglets, has emerged as a model organism for clarifying the processes by which a single probiotic strain can simultaneously influence intestinal barrier integrity, mucosal immunity, and systemic inflammatory responses [28]. This section synthesizes the current understanding of the immune-modulatory mechanisms of N5 and situates these insights within the broader transition from probiotics to postbiotics. Fonseca et al. [29] indicated that oral *Lactobacillus johnsonii* supplementation alleviated respiratory syncytial virus (RSV) infection in mice by altering circulating metabolic profiles and reducing airway Th2 cytokines, impairing dendritic cell function and enhancing regulatory T cell activity.

Although increasing evidence highlights the protective role of certain probiotics against allergen-induced epithelial damage, the exact mechanisms by which *Lactobacillus johnsonii* N5 supernatant (N5sup) mitigates HDM-induced nasal epithelial injury remain largely unexplored. The correlation between the protective attributes of N5 and the inhibition of this UPR pathway is unclear, as it is unclear if additional signaling pathways contribute to its noted benefits. A mechanistic gap exists regarding the regulation of the NR4A1–IRE1–XBP1 axis by a probiotic-derived supernatant to alleviate UPR-induced damage in RPMI-2650 exposed to HDM. This study aims to investigate whether N5sup protects RPMI-2650 from HDM-induced injury and to elucidate the related molecular mechanisms.

## 2. Materials and Methods

### 2.1. Preparation of Lactobacillus johnsonii N5 Supernatant (N5sup)

*Lactobacillus johnsonii* N5 (N5), originally isolated in our laboratory and deposited at the China Center for Type Culture Collection (CCTCC, NO: M2023104) [28], was cultured in MRS broth (Oxoid) at 37 °C for 24 h under anaerobic conditions. Bacterial cells were harvested at the early stationary phase by centrifugation (5000 rpm for 10 min), washed twice with phosphate-buffered saline (PBS), then concentrated 100-fold in a cryoprotective solution containing 0.82 g K_2_HPO_4_, 0.18 g KH_2_PO_4_, 0.59 g sodium citrate, 0.25 g MgSO_4_·7H_2_O, and 172 mL glycerol (87%) made up to 1000 mL with distilled water, and stored at −80 °C. To prepare N5sup, N5 cultures were centrifuged (5000 rpm for 10 min), and the resulting cell-free supernatant was filter-sterilized through 0.22 μm pore-size membranes (Millipore, Billerica, MA, USA). The filtered N5sup was immediately flash-frozen in liquid nitrogen and stored at −80 °C. Untargeted metabolomics analysis of small-molecule metabolites in N5sup was performed, and the results and methods are detailed in our previous report [30]. Data can be found in Supplementary Data S1.

### 2.2. Cell Culture and Treatment

The RPMI-2650 human nasal epithelial carcinoma cell line (BNCC353694) was purchased from BeNa Culture Collection (BNCC, Beijing, China) and cultured in Eagle’s Minimum Essential Medium (EMEM) supplemented with 10% fetal bovine serum (FBS), 100 U/mL penicillin, and 100 μg/mL streptomycin at 37 °C in a humidified incubator with 5% CO_2_.House dust mite (HDM) extract was obtained from Greer Laboratories (XPB82D3A2.5).

**Figure 1:**
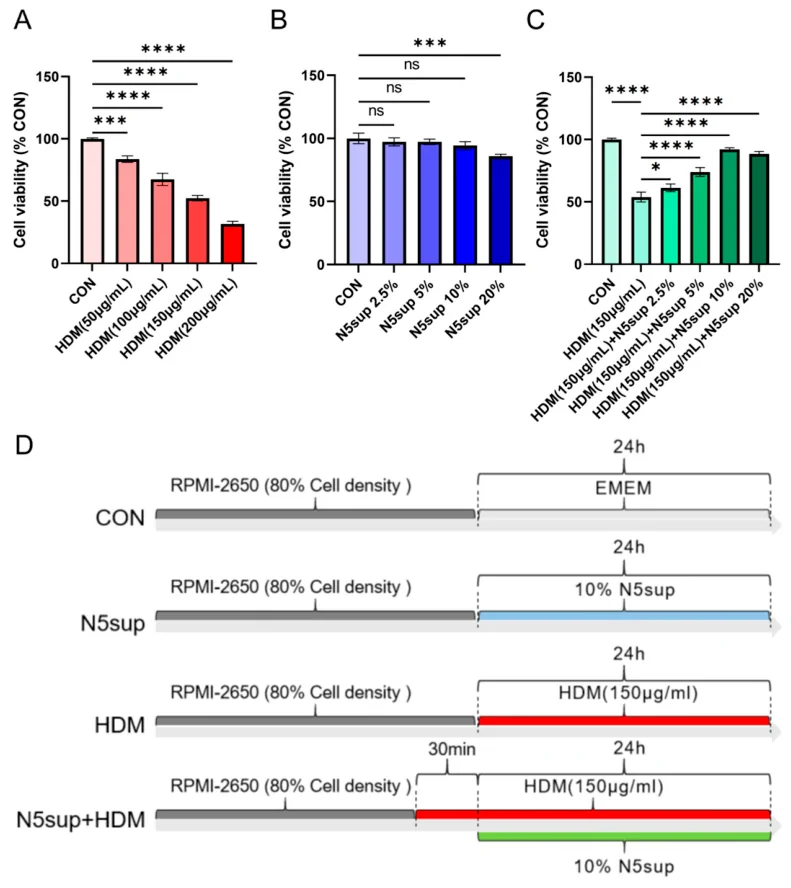
Dose–response screening of HDM and N5sup and schematic illustration of the experimental design. (**A**) CCK-8 assay for cell viability of RPMI-2650 cells treated with gradient concentrations of HDM alone (50, 100, 150, and 200 μg/mL). (**B**) Cell viability detection via CCK-8 assay after single treatment with gradient concentrations of N5sup alone (2.5%, 5%, 10%, and 20% *v*/*v*). (**C**) Cell viability detection following pretreatment with HDM (150 μg/mL) and co-incubation with gradient concentrations of N5sup (2.5%, 5%, 10%, and 20% *v*/*v*). (**D**) Schematic diagram of the experimental design for HDM-induced inflammation in RPMI-2650 human nasal epithelial carcinoma cells. Data are presented as mean ± SEM, *n* = 3. Statistical significance: * *p* < 0.05, *** *p* < 0.001, and **** *p* < 0.0001; ns indicates no significant difference.

To determine the optimal treatment concentrations, dose-finding experiments were performed using the CCK-8 assay (three replicates per concentration). First, cells were treated with HDM at 50, 100, 150, or 200 μg/mL for 24 h; based on cell viability, 150 μg/mL was selected as the inductive concentration (Figure 1A). Second, cells were treated with N5sup alone at 2.5%, 5%, 10%, or 20% (*v*/*v*) for 24 h (Figure 1B). Third, under a fixed HDM concentration of 150 μg/mL, the different concentrations of N5sup were co-incubated with cells for 24 h to compare protective effects (Figure 1C); the results confirmed that 10% N5sup provided the best recovery from HDM-induced damage. Based on these screening results, cells in the formal experiment were randomly divided into four groups: control (normal culture), N5sup (10% N5sup), HDM (150 μg/mL HDM), and N5sup + HDM (pretreatment with 150 μg/mL HDM for 30 min followed by co-incubation with 10% N5sup for 24 h) (Figure 1D). After treatment, cells and culture supernatants were collected for subsequent analyses.

### 2.3. Cell Counting and Cell Counting Kit-8 (CCK-8 Assay)

Cell numbers were counted using a hemocytometer under a light microscope. For the CCK-8 assay (Glpbio Biotechnology, Montclair, CA, USA), cells were seeded in 96-well plates. After treatment, 10 μL of CCK-8 solution was added to each well, and plates were incubated in the dark for 3 h. Absorbance at 450 nm was measured with a microplate reader.

### 2.4. Fluorescence Staining

RPMI-2650 cells were exposed to N5sup, HDM, or N5sup + HDM over a period of 24 h. Following treatment, the medium was eliminated, and the cells were rinsed twice with assay buffer. Subsequently, cells were incubated with calcein (1 µmol/L) at 37 °C for 30 min. Subsequent to incubation, cells were washed twice with phosphate-buffered saline (PBS), secured with a mounting medium, and analyzed using a fluorescence microscope (Olympus Corporation, Tokyo, Japan). For apoptosis detection, the One Step TUNEL Apoptosis Assay Kit (Beyotime Biotechnology, Shanghai, China) was used according to the manufacturer’s instructions. Briefly, cells were fixed, permeabilized, and incubated with the TUNEL reaction solution at 37 °C for 60 min in the dark. After washing, nuclei were counterstained with DAPI. Apoptotic cells exhibited green fluorescence (excitation/emission: 488/530 nm). Images were captured and quantified using ImageJ software (v1.53t). All experiments were performed in three independent biological replicates.

### 2.5. RNA Extraction, Sequencing, and Quantitative RT PCR

Cells from each group (CON, N5sup, HDM, and N5sup + HDM) were seeded separately into one 6-well plate. After treatment, the culture medium was discarded, and cells in each well were lysed with 1 mL of TRIzol (Invitrogen, Waltham, MA, USA). The lysates from all six wells of the same plate were pooled and collected into a single centrifuge tube and thoroughly mixed, and total RNA was extracted. Subsequently, RNA quality was assessed using the Agilent Bioanalyzer 2100 (Agilent Technologies, Santa Clara, CA, USA). High-throughput sequencing was performed by Wuhan Yingzi Gene Technology Co., Ltd. (Wuhan, China) on the BGI T7 (BGI Genomics, Shenzhen, China) sequencing platform. Raw data analysis was carried out using Xshell7 (v7.0.0170p) and Xftp7 (v7.0.0119p), and the FPKM (Fragments Per Kilobase of exon per Million reads mapped) method was employed for quantitative data evaluation [31]. Differential expression analysis between groups was performed using R software (v4.2.0) with the NOISeq method.

Following the manufacturer’s protocol, 1 µg of RNA was reverse-transcribed into cDNA using HiScript II Q RT SuperMix (Vazyme Biotech, R222-01, Nanjing, China). Quantitative qRT-PCR analysis was performed using AceQ qPCR SYBR Green Master Mix (Vazyme Biotech, Q111-02, Nanjing, China) on an ABI QuantStudio 3 Real-Time PCR Instrument (Thermo Fisher Scientific, Waltham, MA, USA). The specific primers used for qRT-PCR were as follows: *ERN1* (forward 5′-GCCACCCTGCAAGAGTATGT-3′, reverse 5′-TTCTCATGGCTCGGAGGAGA-3′), *XBP1S* (forward 5′-TGCTGAGTCCGCAGGGTG-3′, reverse 5′-GCTGGCAGGCTGGGGAG-3′), *NR4A1* (forward 5′-ATGCCCTGTATCCAAGCCCC-3′, reverse 5′-GTGTAGCCGTCCATGAAGGT-3′), and *GAPDH* (forward 5′-TGTTCGTCATGGGTGTGAAC-3′, reverse 5′-ATGGCATGGACTGTGTGTCAT-3′) as the internal reference. Gene expression levels (fold changes) were normalized to *GAPDH* and calculated using the 2^−∆∆CT^ method.

### 2.6. Bioinformatics Analysis of Genes

The Gene Set Enrichment Analysis (GSEA 4.1.0) software was utilized to identify significantly enriched pathways. Additionally, statistical enrichment analysis and classification were conducted on the Gene Ontology (GO) and Kyoto Encyclopedia of Genes and Genomes (KEGG) pathways of the differentially expressed genes (DEGs) utilizing the Metascape database (available online: http://metascape.org/ (accessed on 1 April 2026)) and DAVID (available online: https://davidbioinformatics.nih.gov/ (accessed on 10 April 2026)). Data analysis and visualization were conducted on the online platform (available at http://www.bioinformatics.com.cn, accessed on 16 April 2026), yielding GSEA enrichment analysis plots, KEGG enrichment Sankey diagrams, bubble plots, pathway diagrams, volcano plots, and bar charts of GO-pathway enrichment results. The STRING online functional protein interaction network platform (available at https://cn.string-db.org/ (accessed on 20 April 2026)) was utilized to perform correlation analysis on the differentially expressed genes.

### 2.7. Western Blotting

Cells were lysed in RIPA buffer (Beyotime, P0013B) supplemented with protease and phosphatase inhibitors (Roche, cOmplete™ Mini and PhosSTOP™). Protein concentration was determined using the BCA assay (Thermo Fisher Scientific, Waltham, MA, USA. Equal amounts of protein (30 μg per lane) were separated by 10% SDS-PAGE at 150 V for 60 min and transferred onto PVDF membranes (MilliporeSigma, Burlington, MA, USA) using a wet transfer system at 100 V for 2 h at 4 °C. Membranes were blocked with 5% non-fat milk for 1 h at room temperature. Primary antibodies were incubated overnight at 4 °C with the following dilutions: anti-Occludin (Proteintech, Rosemont, IL, USA, 27260-1-AP, rabbit polyclonal, 1:2000); anti-Claudin-1 (Proteintech, 13050-1-AP, rabbit polyclonal, 1:1000); anti-Claudin-4 (Proteintech, 16195-1-AP, rabbit polyclonal, 1:1000); anti-IRE1 (Proteintech, 27528-1-AP, rabbit polyclonal, 1:1000); anti-NR4A1 (Proteintech, 12235-1-AP, rabbit polyclonal, 1:1000); anti-XBP1S (ABclonal, Woburn, MA, USA, A28350, rabbit polyclonal, 1:2000); and anti-β-actin (Proteintech, 60008-1-Ig, mouse monoclonal, 1:2000). After washing, membranes were incubated with HRP-conjugated secondary antibodies (anti-rabbit or anti-mouse) for 1 h at room temperature. Protein bands were visualized using ECL substrate and imaged with a ChemiDoc system.

### 2.8. Statistical Analysis

Statistical analyses were conducted using the SPSS software package (SPSS version 23, SPSS, Inc., Chicago, IL, USA). A one-way ANOVA was conducted with Tukey’s post hoc analysis. The data were analyzed with GraphPad Prism 9 software (v9.0, GraphPad Software, Inc., San Diego, CA, USA), including multiple comparisons among groups. The data were expressed as the mean and standard error of the mean. Differences were deemed statistically significant at *p* < 0.05.

## 3. Results

### 3.1. N5 Supernatant Alleviates HDM-Induced Reduction in RPMI-2650 Cell Viability

**Figure 2:**
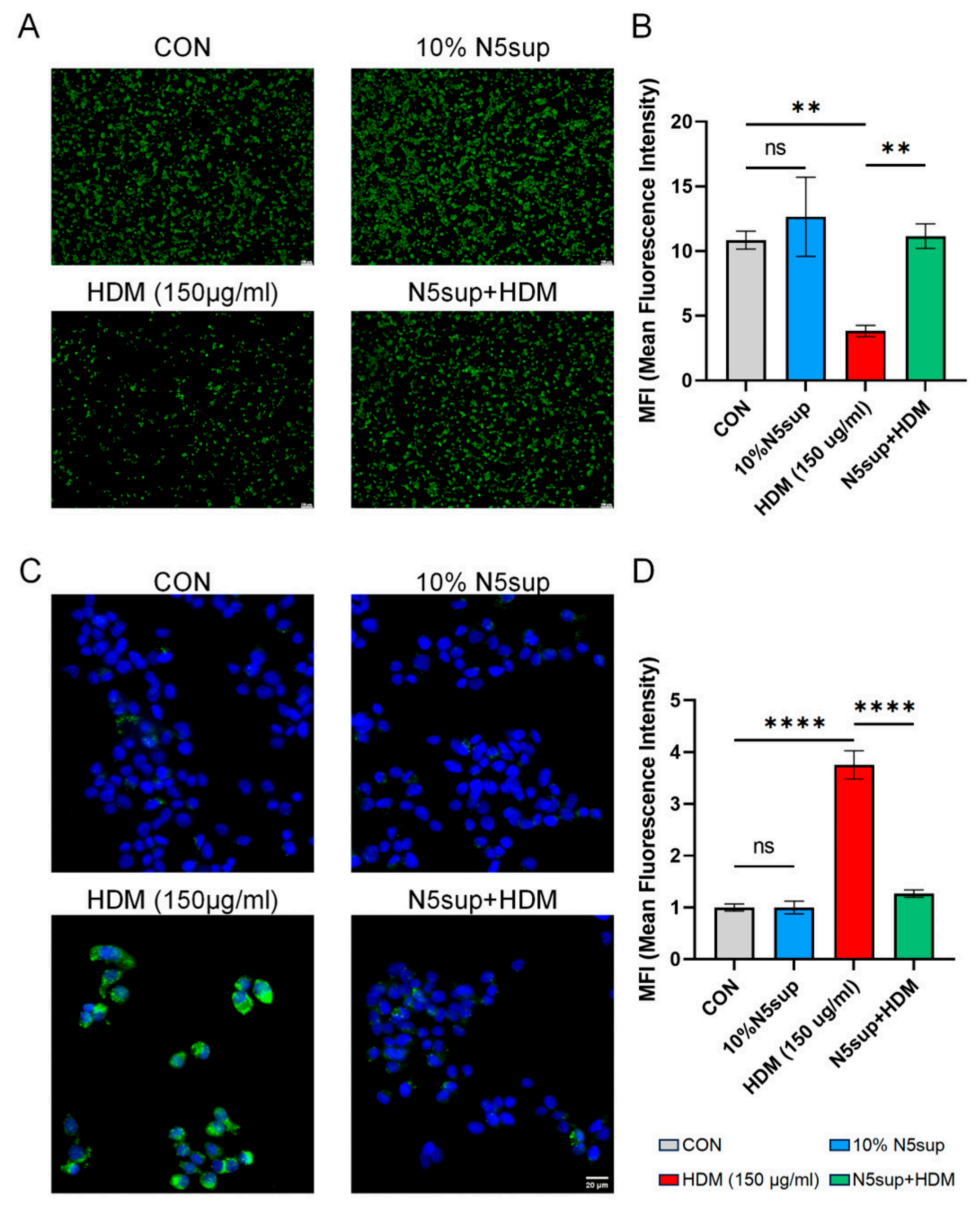
N5sup, HDM, and N5sup + HDM-treated RPMI-2650 cells. (**A**) Calcein staining of cells treated with N5sup, HDM, and N5sup + HDM for 24 h (scale bar = 100 µm). (**B**) Relative fluorescence intensity of RPMI-2650 cells counted after 24 h. (**C**) TUNEL staining of apoptotic cells (green fluorescence) in the CON, N5sup, HDM, and N5sup + HDM groups; nuclei were counterstained with DAPI (blue) (scale bar = 100 µm). (**D**) Quantification of TUNEL-positive cells (mean fluorescence intensity) in each group.Data were presented as the means ± SEM. Statistical significance is considered as follows: ns means not significant, ** *p* < 0.01, and **** *p* < 0.0001; ns indicates no significant difference.

To assess potential protective effects, RPMI-2650 cells treated with N5sup, HDM, and N5sup + HDM underwent live cell staining assays. Live cell staining revealed that, compared with the CON group, N5sup treatment alone for 24 h did not significantly affect cell number (*p* > 0.05), whereas HDM treatment significantly reduced cell number (*p* < 0.05). The addition of N5sup to HDM (N5sup + HDM) resulted in a significant increase in RPMI-2650 cell count compared to the HDM group (*p* < 0.05) (Figure 2A,B). To evaluate whether N5sup protects against HDM-induced cell apoptosis, TUNEL staining was performed. As shown in Figure 2C, HDM treatment significantly increased the number of TUNEL-positive cells (green fluorescence) compared to the CON group, indicating elevated apoptosis (*p* < 0.05), while N5sup alone had no significant effect on apoptosis (*p* > 0.05). In contrast, co-treatment with N5sup markedly reduced the percentage of TUNEL-positive cells relative to the HDM group (*p* < 0.05) (Figure 2D), suggesting that N5sup effectively alleviates HDM-induced apoptosis in RPMI-2650 cells.

### 3.2. Transcriptomic Profiling Identifies ER Stress as a Key Pathway Modulated by N5 Supernatant

**Figure 3:**
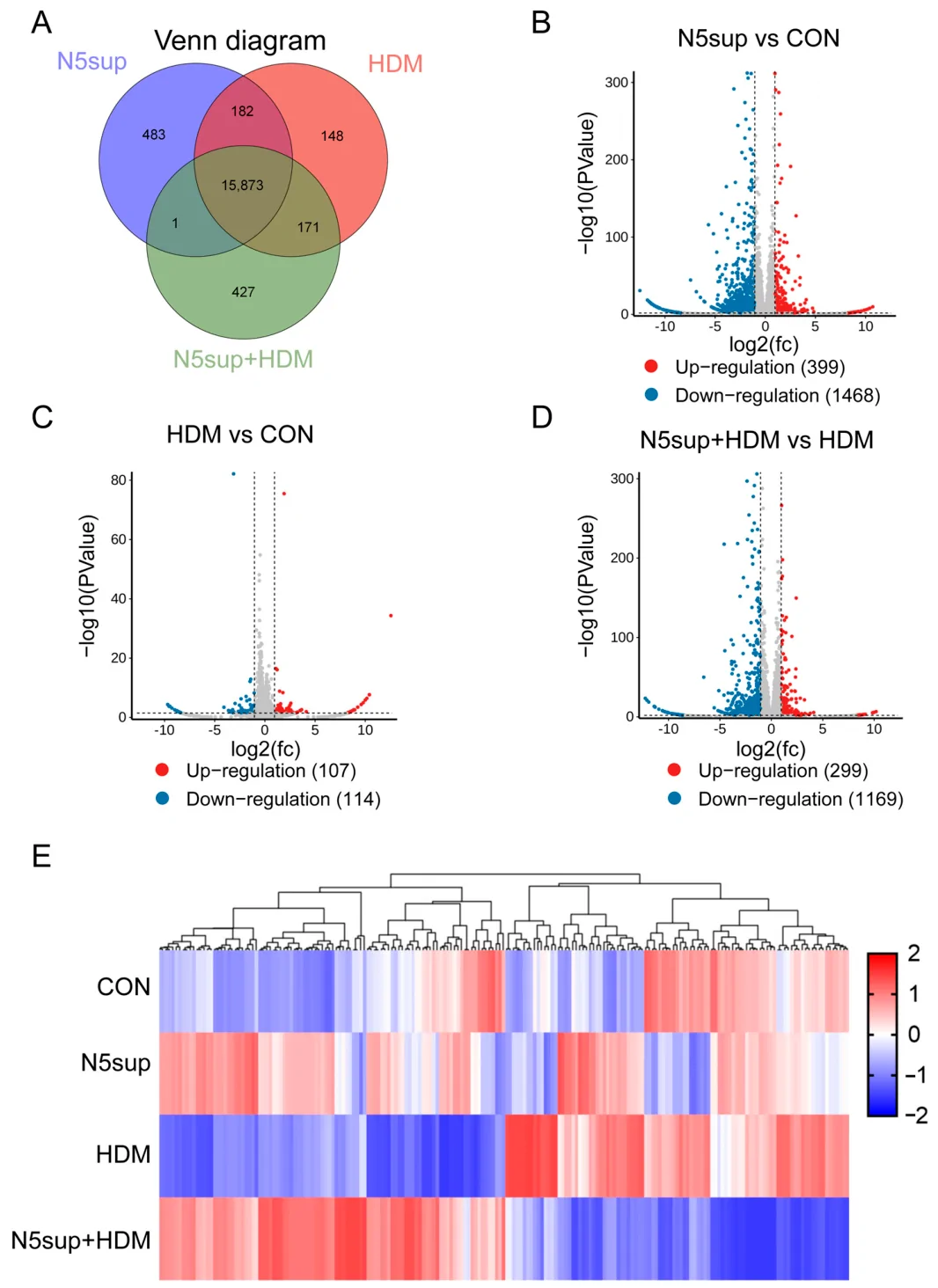
Differential gene expression analysis among experimental groups. (**A**) Venn diagram illustrating the overlap of DEGs across different comparisons. (**B**–**D**) Volcano plot showing the distribution of DEGs, with red and blue dots indicating significantly upregulated and downregulated genes, respectively, and gray dots representing non-significant genes. (**E**) Heatmap of the top 200 DEGs based on fold change, showing relative expression patterns across samples.

To examine the protective mechanism of N5sup against HDM-induced nasal epithelial injury, RNA sequencing was conducted on RPMI-2650 cells under four conditions: CON, N5sup alone, HDM alone, and N5sup + HDM co-treatment. The Venn diagram illustrated distinct and overlapping differentially expressed genes (DEGs) across the comparisons. Significantly, 221 DEGs were exclusively affected by HDM compared to CON, whereas 483 genes were co-regulated in both N5sup against CON and HDM vs. CON comparisons, indicating a fundamental transcriptional response influenced by N5sup (Figure 3A). Volcano plots (−log10 (*p*-value) vs. log2 (fold change)) for each comparison indicated substantial gene overexpression (red) and downregulation (blue). The HDM challenge elicited extensive transcriptional alterations, but N5sup therapy alone exhibited a restricted number of differentially expressed genes (DEGs). Co-treatment with N5sup + HDM altered the expression pattern to resemble the CON profile, suggesting a restorative impact (Figure 3B,D). The heatmap of the top 200 DEGs ordered by fold change exhibited distinct clustering of biological replicates and inverted expression patterns in the N5sup + HDM group relative to HDM alone (Figure 3E). Gene enrichment analysis associated these alterations with the unfolded protein response (UPR), specifically implicating *NR4A1*, *IRE1*, and *XBP1*. The data collectively indicate that N5sup safeguards nasal epithelial cells from HDM-induced damage by inhibiting the NR4A1–IRE1–XBP1-mediated UPR pathway.

**Figure 4:**
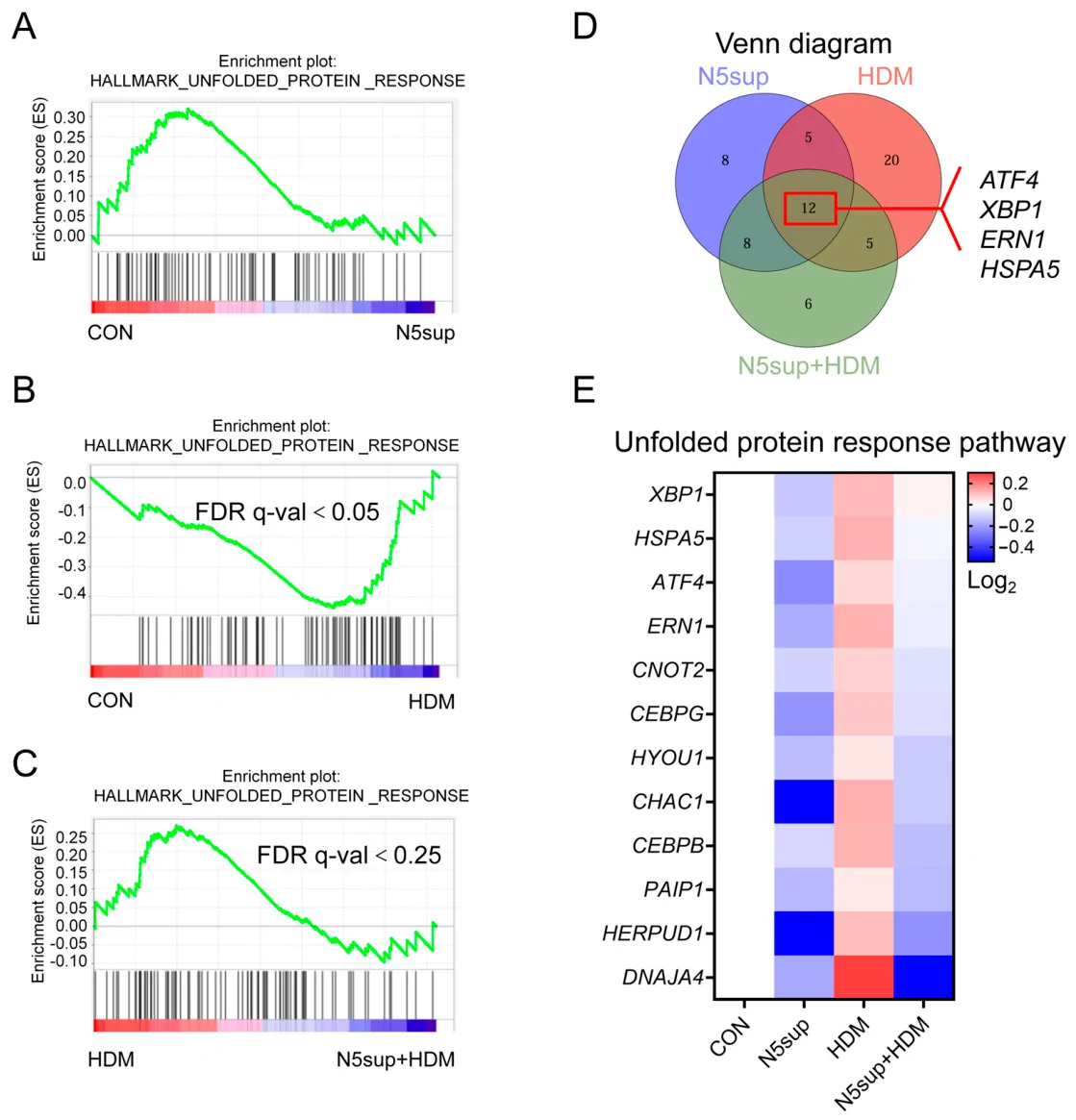
N5sup attenuates HDM-induced activation of the unfolded protein response pathway. (**A**–**C**) GSEA enrichment plots of the unfolded protein response pathway in the indicated comparisons. (**D**) Venn diagram showing the overlap of core enrichment genes among the three comparisons. (**E**) Heatmap illustrating the relative expression of shared genes identified in the Venn diagram across all samples. FDR = false discovery rate.

Gene Set Enrichment Analysis (GSEA) was conducted to ascertain if N5sup modifies the UPR in HDM-challenged RPMI-2650 cells. Enrichment plots for the hallmark unfolded protein response gene set demonstrated that HDM alone significantly enriched this pathway (FDR q-value < 0.05) (Figure 4B), while N5sup therapy alone exhibited no enrichment relative to CON (Figure 4A). Significantly, co-treatment with N5sup + HDM substantially diminished UPR enrichment relative to HDM alone (FDR q-val < 0.25) (Figure 4C), suggesting a protective impact. The Venn map of core enrichment genes across comparisons revealed essential UPR regulators specifically common to HDM vs. CON and N5sup + HDM vs. HDM (Figure 4D). Among these, *ATF4*, *XBP1*, *ERN1*, and *HSPA5* were pivotal nodes. The heatmap illustrated the relative expression of these common genes across all experimental groups (CON, N5sup, HDM, and N5sup + HDM) (Figure 4E). The HDM challenge elicited a significant elevation of *XBP1*, *ATF4*, *ERN1*, and their downstream effectors (*HERPUD1* and *CHAC1*). Significantly, co-treatment with N5sup + HDM mitigated this trend, decreasing expression to control levels. The results indicate that N5sup safeguards against HDM-induced epithelium damage by directly obstructing the NR4A1–IRE1–XBP1-mediated UPR pathway.

### 3.3. N5sup Suppresses the NR4A1–IRE1–XBP1 Signaling Axis

**Figure 5:**
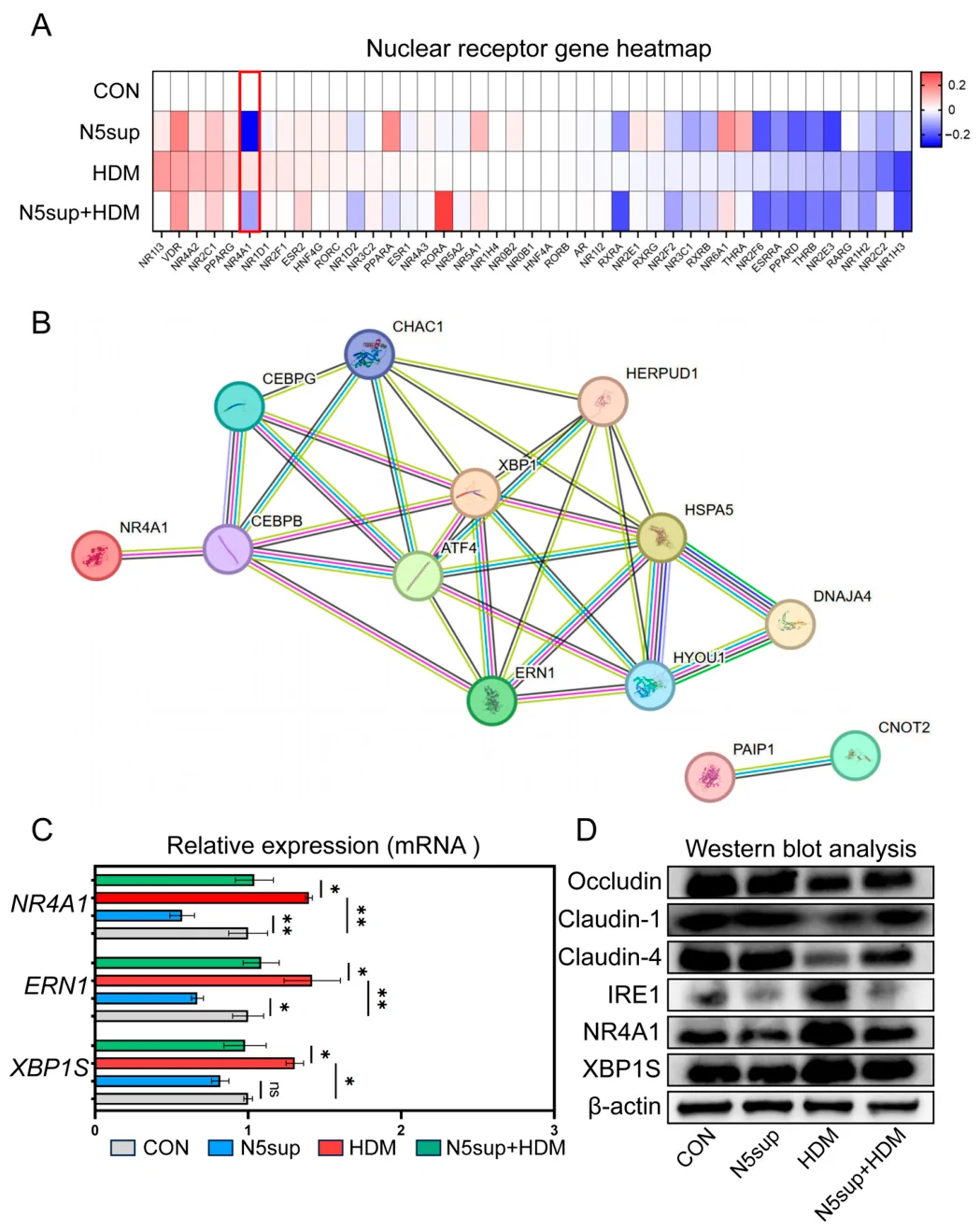
N5sup suppresses HDM-induced activation of the NR4A1–IRE1–XBP1 signaling axis. (**A**) Heatmap illustrating the expression patterns of nuclear receptor-related genes across experimental groups. (**B**) Protein–protein interaction network of selected nuclear receptor-related genes generated using the STRING database. (**C**) Relative mRNA expression levels of *NR4A1*, *ERN1* and *XBP1S*. (**D**) Western blot analysis of Occludin, Claudin-1, Claudin-4, IRE1, NR4A1, XBP1s and β-actin protein expression. Statistical significance is considered as follows: ns means not significant, * *p* < 0.05, and ** *p* < 0.01; ns indicates no significant difference.

To clarify the molecular mechanism by which N5sup mitigates the UPR, we concentrated on nuclear receptor family genes involved in UPR regulation. The heatmap depicting expression patterns of nuclear receptor-related genes across the CON, N5sup, HDM, and N5sup + HDM groups demonstrated that HDM challenge significantly altered several nuclear receptor genes, including *NR4A1*, *ATF4*, *XBP1*, *ERN1*, *CHAC1*, and *HERPUD1*. Co-treatment with N5sup + HDM reinstated these genes, returning expression levels similar to the CON group. N5sup alone exhibited negligible effects (Figure 5A). The protein–protein interaction network produced from the STRING database revealed a densely linked hub focused on *NR4A1*, *IRE1*, and *XBP1*, with further associations to ATF4, HSPA5, and CHAC1. This network facilitates a coordinated signaling pathway in which NR4A1 functions upstream of the *IRE1*–*XBP1* branch of the unfolded protein response (Figure 5B). Quantification of relative mRNA expression via qRT-PCR demonstrated that HDM markedly elevated transcript levels of *XBP1*, *ERN1*, and *NR4A1* in comparison to CON. N5sup + HDM co-treatment significantly diminished these elevations (Figure 5C). Western blot analysis further corroborated these transcriptional changes and simultaneously assessed the expression of epithelial barrier-associated tight junction proteins. Notably, N5sup alone did not significantly alter the protein levels of Occludin, Claudin-1, and Claudin-4; however, HDM stimulation markedly reduced their expression, an effect that was effectively counteracted by N5sup co-treatment (Figure 5D). Concurrently, the HDM-induced upregulation of IRE1, NR4A1, and the active spliced variant XBP1s was also markedly attenuated by N5sup co-treatment (Figure 5D). Collectively, these data indicate that N5sup protects RPMI-2650 cells from HDM-induced injury, potentially through targeting the NR4A1–IRE1–XBP1 signaling axis.

## 4. Discussion

House dust mites (HDMs) are a primary source of indoor aeroallergens that contribute substantially to allergic rhinitis, asthma, and atopic dermatitis, primarily through their protease-mediated disruption of the airway epithelial barrier and subsequent induction of type 2 inflammatory responses [32,33]. The allergens of HDM are mainly present in the mites’ bodies and their metabolic residues, such as feces, and their composition is remarkably complex. At least 20 distinct allergens have been recognized so far, including *Der p* 1, *Der f* 1, *Der p* 2, and *Der f* 2 [34,35]. In addition, HDM itself carries intrinsic pro-inflammatory agents, including endotoxins and bacterial DNA, which may also contribute to its overall pathogenicity [36,37]. Although probiotic-based interventions for allergic airway diseases have garnered growing interest, the molecular mechanisms by which probiotic-derived products protect nasal epithelial cells against HDM-induced injury remain incompletely defined. *Lactobacillus johnsonii*, a representative intestinal probiotic, is extensively found in the gastrointestinal tracts of several hosts, including humans, mice, dogs, birds, pigs, and honeybees [38,39], and has a long history of application in the food and fermented feed industries [40]. In the present study, we demonstrate that the cell-free *Lactobacillus johnsonii* N5 supernatant (N5sup) effectively protects RPMI-2650 human nasal epithelial carcinoma cells from HDM-induced damage, and that this protection is associated with suppression of the NR4A1–IRE1–XBP1-mediated unfolded protein response (UPR) pathway.

A key observation from our transcriptomic and biochemical analyses is that HDM challenge robustly activates the IRE1-XBP1-mediated UPR branch, as evidenced by significant enrichment of the hallmark unfolded protein response gene set and upregulation of *ERN1* (encoding IRE1), *XBP1*, and their downstream effectors *CHAC1* and *HERPUD1*. This is consistent with recent evidence that allergen-induced ER stress leads to apoptosis in airway epithelial cells [41]. Notably, the IRE1–XBP1 branch of the UPR has been implicated in the pathogenesis of allergic asthma and chronic rhinosinusitis, where sustained ER stress promotes pro-inflammatory cytokine production and facilitates epithelial–immune cell crosstalk [42,43]. Our data extend these observations by demonstrating that HDM-induced UPR activation in nasal epithelial cells is accompanied by reduced expression of tight junction proteins (Occludin, Claudin-1, and Claudin-4) and increased apoptosis. Previous studies have also shown that HDM treatment significantly reduces transepithelial electrical resistance (TEER) in airway epithelial cells, accompanied by altered expression and localization of tight junction proteins such as Occludin and Claudin-1 [44,45], which is partially consistent with our findings; however, our assessment of the barrier-protective function of N5sup remains limited. Importantly, co-treatment with N5sup reversed all of these HDM-induced changes, indicating that the protective effect of N5sup is at least partly mediated through attenuation of the IRE1–XBP1-mediated UPR pathway.

Unlike previous reports, our study has identified the orphan nuclear receptor NR4A1 as being potentially associated with the IRE1–XBP1 axis in this context. NR4A1, or Nur77, is part of the NR4A subfamily, which encompasses NR4A2 (Nurr1) and NR4A3 (NOR-1, MINOR), and functions as an orphan nuclear receptor with an unidentified natural ligand. Members of the NR4A subfamily typically operate by regulating gene expression in response to external stimuli. Its designation as an “immediate early gene” that responds to various cellular stresses suggests it may serve as a potential integrator of ER stress signaling. *NR4A1* expression was notably detected among differentially expressed genes in lung epithelial cells after HDM exposure in single-cell RNA-seq analysis [46,47], suggesting it is transcriptionally regulated in the context of allergen-induced epithelial perturbation. The *Tet1*-dependent epigenetic modulation of HDM responses in epithelial cells suggests that transcription factor networks, presumably involving NR4A family members, are restructured during allergic inflammation [48]. Our findings suggest that NR4A1 may function as a stress-responsive transcriptional regulator that amplifies IRE1–XBP1 signaling upon HDM stimulation. This notion is supported by previous studies showing that NR4A1 can modulate ER stress in pancreatic cancer cells [20] and that its expression is altered in inflamed tissues [22]. However, whether NR4A1 directly binds to and activates the IRE1–XBP1 pathway remains an open question that warrants future investigation.

IRE1α, expressed by the *ERN1* gene, is a type I transmembrane protein featuring two catalytic domains: a serine/threonine kinase domain and an endoribonuclease (RNase) domain [49,50,51,52]. It participates in various cellular processes and governs both cell survival and apoptosis. The activated IRE1 excises a 26-nucleotide intron from the coding sequence of the transcription factor *XBP1* [53,54,55]. This atypical splicing event causes a shift in the coding reading frame of the mRNA, resulting in the production of a more stable and active transcription factor, designated *XBP1s*, which also signifies the activation of the UPR [56]. Subsequently, XBP1s translocates to the nucleus and binds to the promoters of its target genes, including those encoding molecular chaperones and proteins involved in ER-associated degradation. In the context of HDM-induced nasal epithelial damage, the IRE1α–XBP1 axis has been implicated in multiple deleterious processes when ER stress becomes chronic or unresolved. Studies have shown that XBP1 stimulates the production of pro-inflammatory mediators and facilitates epithelial–immune cell interactions, particularly in the regulation of epithelium–dendritic cell communication—a finding consistent with our observations. However, whether the IRE1α–XBP1 axis in our model is directly mediated by NR4A1 remains unclear; our study proposes this possibility based on the functional characteristics of nuclear receptors.

An intriguing but still speculative aspect of our work concerns the active components within N5sup that mediate these effects. Previous untargeted metabolomic profiling of the same N5sup identified several bioactive metabolites, including 3-phenyllactic acid, N-acetylornithine, and DL-lactic acid [30]. Among these, 3-phenyllactic acid has demonstrated antioxidant and anti-inflammatory [57] properties in other experimental systems. We speculate that one or more of these metabolites may contribute to the UPR-suppressive activity observed here, either by directly modulating ER stress sensors or by attenuating upstream oxidative or inflammatory signals that drive UPR activation. Nevertheless, this hypothesis remains tentative, and systematic fractionation of N5sup followed by activity-guided identification is required to pinpoint the precise molecular entities responsible for the observed protection.

Several limitations of this study should be acknowledged. First, all experiments were performed in an immortalized human nasal epithelial cell line, which may not fully recapitulate the complex multicellular and immune environment of the native nasal mucosa. Moreover, the experimental design is based on a therapeutic model, which has certain limitations; future studies will also focus on the preventive effect of N5 supernatant against HDM. Second, although we observed strong correlations among NR4A1, IRE1, and XBP1 expression, the causal hierarchy and the exact molecular events linking NR4A1 to IRE1–XBP1 activation remain to be experimentally defined. Third, the specific active components in N5sup responsible for inhibiting the NR4A1–IRE1–XBP1 pathway have yet to be determined. Finally, the applicability of this protective mechanism to other allergens or different types of respiratory epithelial cells requires further validation and has not been addressed in the current study, representing a critical direction for future investigation.

## 5. Conclusions

This study concludes that N5sup effectively protects RPMI-2650 human nasal epithelial carcinoma cells against HDM-induced injury, and that this protective effect is associated with suppression of the NR4A1–IRE1–XBP1-related unfolded protein response (UPR) pathway. HDM challenge significantly activated this signaling axis, accompanied by extensive transcriptional dysregulation and marked enrichment of the UPR pathway. Co-treatment with N5sup reversed these effects, reducing the expression of key UPR mediators—including NR4A1, IRE1, and the active spliced variant XBP1s—at both transcriptional and translational levels. Collectively, these findings suggest that the NR4A1–IRE1–XBP1 pathway may serve as an important regulatory node in HDM-induced epithelial damage and highlight N5sup as a potential postbiotic intervention for allergic airway conditions, including allergic rhinitis. Future studies are warranted to explore the clinical translational potential of this strain and to systematically identify the specific bioactive components within N5sup that confer its protective properties.

## Data Availability

The raw RNA-seq data are publicly available in the NCBI BioProject database under accession number PRJNA1497102.

## Supplementary Materials

The following supporting information can be downloaded at https://www.mdpi.com/article/10.3390/metabo16090634/s1. Supplementary Data S1: Untargeted metabolomics.

## Abbreviations

The following abbreviations are used in this manuscript:

Abbreviation	Full Term
ATF4	Activating transcription factor 4
ATF6	Activating transcription factor 6
BiP	Binding immunoglobulin protein
CCK-8	Cell Counting Kit-8
CHAC1	ChaC glutathione-specific gamma-glutamylcyclotransferase 1
DEG	Differentially expressed gene
ER	Endoplasmic reticulum
ERAD	ER-associated degradation
ERN1	Endoplasmic reticulum to nucleus signaling 1 (gene encoding IRE1)
FC	Fold change
FDR	False discovery rate
FPKM	Fragments Per Kilobase of exon per Million reads mapped
GIT	Gastrointestinal tract
GO	Gene Ontology
GSEA	Gene Set Enrichment Analysis
HDM	House dust mite
HERPUD1	Homocysteine-responsive endoplasmic reticulum-resident ubiquitin-like domain member 1
HRP	Horseradish peroxidase
IRE1	Inositol-requiring enzyme 1
KEGG	Kyoto Encyclopedia of Genes and Genomes
N5	*Lactobacillus johnsonii* N5
N5sup	*Lactobacillus johnsonii* N5 supernatant
NR4A1	Nuclear receptor subfamily 4 group A member 1
PBS	Phosphate-buffered saline
PERK	PKR-like ER kinase
qRT-PCR	Quantitative real-time polymerase chain reaction
RNA-seq	RNA sequencing
RSV	Respiratory syncytial virus
SEM	Standard error of the mean
TEER	Transepithelial electrical resistance
Th2	T helper type 2
TJ	Tight junction
TSLP	Thymic stromal lymphopoietin
UPR	Unfolded protein response
XBP1	X-box binding protein 1
XBP1s	Spliced XBP1
XBP1u	Unspliced XBP1 mRNA
